# Ink Animation VR Design Based on Wireless Sensor and HTML5 Interactive Technology

**DOI:** 10.1155/2022/7574231

**Published:** 2022-05-29

**Authors:** Wei Ding

**Affiliations:** School of Journalism & Communication, Anhui Normal University, Wuhu, Anhui 241002, China

## Abstract

Ink element is one of the unique cultural symbols in China, which has profound cultural heritage and spiritual connotation. Animation is a form of art design, and it is a good way to convey and express information in an intuitive artistic image. The use of ink and wash elements for animation design has important value and significance in the start of Chinese culture. It can develop animation design, innovate animation creation forms, and can highlight Chinese style. In this article, we have studied the creation process, creation format, and creation method of using angular digital technology to make ink VR design. It combines art and technology and combines practical experience with wireless sensors. Based on HTML5 interactive technology, we have studied its advantages more deeply, better introduced the theme of ink element animation, solved the problem of realizing ink effect in 3D virtual environment, and better introduced ink animation in virtual reality environment. During the production process, students will learn how to simulate digital objects, render ink and wash effects, build a virtual environment in the later stage, and explore the knowledge, operation, and meaning of ink and wash elements and the animation design process. This paper studies the wireless sensor and HTML5 interactive technology and applies it to the design of ink and wash animation VR, aiming to promote its vigorous development and application.

## 1. Introduction

In the 1960s, ink and wash animation appeared in the public eye and became a unique style of art film. Taking the traditional Chinese ink and wash sketch method as the main modeling expression method, the image and ink moving on the screen use video to generate photographic frames. Typical examples are traditional ink and wash animations, such as “Little Tadpoles Looking for Mother” and “Landscape.” At that time, Chinese animation was very prominent. It is simple, elegant, and poetic, and is loved by the public for its fresh and elegant artistic expression. It has unique characteristics in every animation field in Japan and abroad [1]. However, the creation process of traditional ink and wash animation is very complicated. Due to the exhaustion of material and spiritual costs such as financial and human resources, the production of animation is very different from the commercial market. Chinese ink and wash animation is slowly disappearing and gradually becoming a bystander. With the advent of the digital age and the rapid development of computer technology, major changes have taken place in artistic animation creation methods, and artistic animation ink creation has also obtained VR design and new development opportunities [2]. Animation ink realizes a digital platform that organically combines computer technology and elemental ink, making ink and wash animation more expressive. Digital ink and wash animation is very different from traditional ink and wash animation in terms of production methods, as well as different expression methods and creative purposes [3]. In 2016, virtual reality technology came to us with a strong attitude. With the support of three-dimensional computer technology and wireless sensors, virtual reality technology can create a virtual environment for us [4]. It is three-dimensional, lifelike, and has continuous vision, hearing, touch, smell, and taste perception capabilities, so people can be natural. The ground interacts with the objects in the virtual environment. Each object has a sense and experience equivalent to realism, so the corresponding VR animation was born [5]. The virtual world gave us a new world of traditional animation. In the big environment of digital video ink painting based on HTML5 interactive technology, interactive technology will get greater development with technical support [6]. How to adapt to the aesthetics of modern people and how to construct Chinese characteristics in the creative video is worthy of in-depth thinking and is a research problem. This article aims to explore and study the actual production of three-dimensional ink and wash animation in the VR virtual reality environment from the connection between theory and practice [7]. We briefly publish related concepts and theories, then link them with our own practical experience, focusing on how to make VR ink and wash animations, and analyze the implementation methods and routes of related 3D software and VR animation technology [8].

## 2. Related Work

The literature introduces the evaluation criteria of the network's own positioning system and algorithm performance in the sensor system, combines effective classification methods and corresponding research content, combined with representative methods for measuring network sensing distances, and analyzes the calculation of nodes [9]. Fully combine the calculated data to start the simulation. The literature describes a variety of algorithms currently in use. On the basis of the original typical positioning technology, the algorithm is improved and the two-dimensional algorithm is extended to the three-dimensional space [10]. That is, the focus is on a three-dimensional, fast, and accurate positioning algorithm based on RSSI non-ranging. This positioning technology can be used under the premise of greatly reducing hardware costs and communication costs, and relatively high positioning accuracy can be obtained [11]. The literature describes the positioning technology of the sensor. Based on the introduction of the basic theory and terminology of positioning, combined with the key technical theoretical content, through the ranging technology, the application of the rationalization algorithm for the distance is realized [12]. The literature introduces the practical application of typical research and analysis theories, combined with error analysis of positioning algorithms, based on the revised RSSI value. Also, it can be combined with the corresponding MATLAB to carry out simulation research and application of the algorithm, and rationalize and evaluate the research and analysis content of the distance measurement value in the corrected state and the related error in the traditional model system structure and promote the related problems in the error system structure [13]. The literature introduces a type of noise-immune wireless sensor network node location algorithm (noise-immune LoCalization algorithm via low-rank Matrix Decomposition, LoCMD). This algorithm introduces the popular low-rank matrix factorization technology in the field of machine learning, which can complement the missing parts in the ranging information, avoiding the problem of limited scalability caused by solving the kernel norm minimization matrix complement problem; at the same time, use the Gaussian mixture distribution to fit the unknown noise type in the ranging information in the complex environment [14].

## 3. Wireless Sensors and HTML5 Interactive Technology

### 3.1. Wireless Sensor

**Figure 1:**
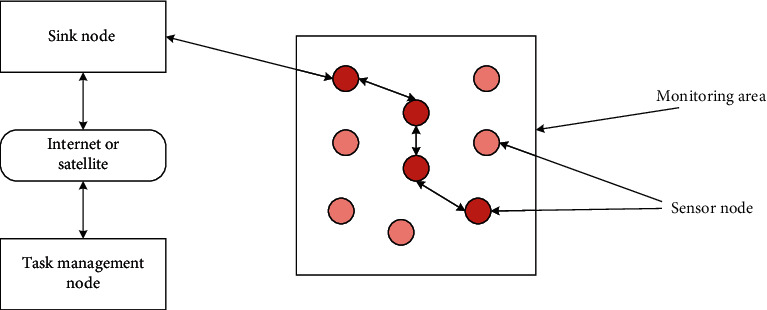
Wireless sensor network architecture.

The main system construction diagram of the wireless sensor network device is composed of the following parts (Figure 1).

In the process of research and innovation, people focus on WSN, which can be combined with aircraft to achieve spreading. At the same time, it can also be combined with manual distribution to transmit or arrange to all corners of the monitoring area. By composing different nodes, the overall network can be realized. Corresponding sensor nodes can analyze the aggregation nodes through the transmission of different node systems to analyze the relevant sensing information content and data fusion of different nodes and can use satellite systems to realize the rationalized application of management nodes, and through the task supervision of the monitoring person, the feasibility analysis of each monitoring task and node data is promoted [15].

1. *Sensor Node*. Usually, the sensor node system and structure are embedded, and the battery is used to provide the corresponding power. In the calculation process of sensor nodes, the feasibility analysis of data storage function and corresponding communication ability should be carried out to realize the continuous application of node communication structure and communication theory. In the process of data exchange, based on the data exchange of peripheral nodes, the feasibility analysis of the data of the whole network node is realized, and the storage management mode is combined with the structure of fusion data to jointly complete the data information and explore the realized functions.
2. *Convergence Node*. In the process of converging nodes, it can improve related computing capabilities through more powerful computing functions and computing systems. It can be combined with the external network architecture of WSN to promote the feasibility analysis of communication network protocols between protocol stacks, and can be combined with effective broadcasting to transmit the corresponding information structure between the Internet in time. At the same time, it should be combined with the communication function system to realize the rational construction of the overall sensor node network system and instruments.
3. *Task Management Node*. It can combine dynamic wireless network sensor structure and functions to realize effective analysis of node management and promote the rational construction of wireless network sensor network resources.

### 3.2. HTML5 Interactive Technology

#### 3.2.1. The Design Concept of HTML5

HTML5 is a digital product based on CSS, JS, and other computer languages and integrated with multi-dimensional audio-visual special effects. HTML5 is a more high-end parent that can contain all branches, not a string of codes, a symbol, or a slideshow per web page. Its design allows information to be disseminated more quickly and efficiently, which not only reduces the cost of operators but also enhances the dissemination and click-through rate of information.

The emergence of HTML5 has brought new breakthroughs to the original design environment. Due to its unique browsing method and development background, it has reduced operating costs and entrepreneurial risks for many companies and individual designers. Not only that, the emergence of HTML5 has also increased user coverage and participation. It is also called hypermedia because it can be used for dynamic interaction on the mobile phone. Hypermedia contains all content except text, such as pictures, music, video, programming, etc. These non-text contents are called “hypertexts.” When they are “marked,” they need to be decoded to integrate all attributes.

HTML5 is not just a “hypertext” and “markup” language. It provides more possibilities for the development and spread of the Internet today, and it also provides a more tolerant and more diverse Internet for many operators and designers.

#### 3.2.2. HTML5 Interactive Design

*(1) HTML5 Interface Interaction*. Interaction design, as the name implies, refers to the design of mutual communication and interaction between objects. The main content of interaction design is how to make users accurately accept and understand platform operations and is supplemented by a humanized interaction experience, which is more concise and makes the information transmitted by users more intimate and cordial.

Compared with other platforms, HTML5 can implement many complex functions without plug-ins, and users can perform tasks more easily. Although HTML5 can be used across platforms, there are certain restrictions on the display screen. Compared with the size of the computer screen, the interface of a mobile device can be touched for very limited interaction during use. Therefore, when designers are designing HTML5, the focus is to allow users to quickly understand information. Abandon the functions and unnecessary operations in the interface and focus on the functional items of the software, so that all user operations can be responded to and accurate and concise interface interactions are provided to users to provide the best user experience.

In addition to text and pictures, the content of interactive design also includes audio-visual language and touch interaction. Excellent interaction design needs to stand on the user's standpoint, design the product more efficiently and concisely, and strengthen the user-friendly design during the user's use. Therefore, designers need to empathize when designing, first to explore the needs and feelings of users and to guess the reactions and emotions of users during use. By coordinating the delicate balance between application software and users, the two are interdependent. Allow users to complete their own needs through application software, and the software can also be continuously upgraded through users.

#### 3.2.3. Advantages and Disadvantages of HTML5

This topic is mainly presented in the form of HTML5. As a new Internet web development standard, HTML5 is welcomed by more and more users. Compared with other platforms, the biggest advantage of HTML5 is compatibility. It is suitable for all groups of people. Different mobile terminals and systems can be used for browsing, which minimizes usage restrictions.

The target group for this topic is young people, who are quick to learn, are willing to accept fresh objects, and like to use high-tech products. Therefore, reducing the burden of use and quickly implementing orders are their main demands. For them, HTML5 has a better user experience. With the current vigorous development of online social networking, it has undoubtedly brought huge inconvenience to many users. Because the compatibility between the two platforms is too poor, most companies need to use two development teams when designing and developing applications. According to the characteristics of different systems, the development and shelf nodes often need to be staggered before and after. It will affect the work efficiency between the teams and the company's economic benefits and at the same time bring a mental gap to users on different platforms.

Based on the above situation, HTML5 has the following advantages.

1. HTML5 has strong compatibility.
2. Compared with the application, its startup time is shorter, the Internet connection speed is faster, it does not need to be downloaded in the software store, does not occupy storage space, and can be used simply by scanning the code. It is especially suitable for mobile devices such as mobile phones and tablets.
3. Ultra-high detection efficiency gives suppliers a great advantage in product promotion.
4. HTML5's unique real-time update and excellent differential update experience also greatly reduce its barriers to use. In the last five years, HTML5 has been widely used in various industry media.

However, HTML5 technology also has several problems. First of all, in terms of animation, animation in HTML5 consumes more resources and has fewer types. Compared with app, it is more than single. In addition, HTML5 obtains data through the process of obtaining, loading, and displaying. The complete display of the interface requires all data to be loaded. This is closely related to network traffic. Once the network speed is too slow, it will directly cause the web page to fail to open or to wait too long. During scene switching, if the cached data occupy most of the memory, the application is also prone to lag, which reduces the user experience.

But despite this, cross-platform use and easy loading methods still make HTML5 the main choice for application vendors and browser vendors.

### 3.3. Data Principle

Among them, in the feasibility study of RSSI, TOA, and corresponding AOA technology, the distance of the corresponding node can be realized on a certain basis, and the relative position of the unknown node can be calculated.

Accordingly,

(1) $$\begin{array}{c} \left\{\begin{array}{c} \sqrt{{\left(x-x_{a1}\right)}^{2}+{\left(y-y_{a1}\right)}^{2}}=d_{a1}\\ \sqrt{{\left(x-x_{a2}\right)}^{2}+{\left(y-y_{a2}\right)}^{2}}=d_{a2}\\ \sqrt{{\left(x-x_{a3}\right)}^{2}+{\left(y-y_{a3}\right)}^{2}}=d_{a3} \end{array}\right.. \end{array}$$

Solving the above formula, the coordinates of the node *s* at the unknown position can be obtained as

(2) $$\begin{array}{c} \left[\begin{array}{c} x\\ y \end{array}\right]={\left[\begin{array}{cc} 2\left(x_{a1}-x_{a3}\right) & 2\left(y_{a1}-y_{a3}\right)\\ 2\left(x_{a2}-x_{a3}\right) & 2\left(y_{a2}-y_{a3}\right) \end{array}\right]}^{-1}\left[\begin{array}{c} x_{a1}^{2}-x_{a3}^{2}+y_{a1}^{2}-y_{a3}^{2}+d_{a1}^{2}-d_{a3}^{2}\\ x_{a2}^{2}-x_{a3}^{2}+y_{a2}^{2}-y_{a3}^{2}+d_{a2}^{2}-d_{a3}^{2} \end{array}\right]. \end{array}$$

Triangulation method:

(3) $$\begin{array}{c} \left\{\begin{array}{c} \sqrt{{\left(x_{c1}-x_{a1}\right)}^{2}+{\left(y_{c1}-y_{a1}\right)}^{2}}=d_{1}\\ \sqrt{{\left(x_{c1}-x_{a3}\right)}^{2}+{\left(y_{c1}-y_{a3}\right)}^{2}}=d_{1}\\ \sqrt{{\left(x_{a1}-x_{a3}\right)}^{2}+{\left(y_{a1}-y_{a3}\right)}^{2}}=2d_{1}^{2}-2d_{1}^{2}\cos\alpha \end{array}\right.. \end{array}$$

From the above formula, the coordinates and radius of the center of the circle can be determined. Similarly, the coordinates and radius of the center of the circle corresponding to *A*, *B*, ∠*ABD* and *B*, *C*, ∠*BDC* can be obtained in the same way. Finally, the coordinates of the target node can be determined by using the trilateration method, that is, the position of the unknown node can be obtained.

It will be composed of the following equations:

(4) $$\begin{array}{c} \left\{\begin{array}{c} {\left(x_{1}-x\right)}^{2}+{\left(y_{1}-y\right)}^{2}=r_{1}^{2}\\ \vdots \\ {\left(x_{m}-x\right)}^{2}+{\left(y_{m}-y\right)}^{2}=r_{m}^{2} \end{array}\right.. \end{array}$$

The first *m* − 1 equation in the above equation is subtracted from the *m*th equation in turn. After sorting, the following equation is obtained:

(5) $$\begin{array}{c} \left\{\begin{array}{c} x_{1}^{2}-x_{m}^{2}-2x\left(x_{1}-x_{m}\right)+y_{1}^{2}-y_{m}^{2}-2y\left(y_{1}-y_{m}\right)=r_{1}^{2}-r_{m}^{2}\\ \vdots \\ x_{m-1}^{2}-x_{m}^{2}-2x\left(x_{m-1}-x_{m}\right)+y_{m-1}^{2}-y_{m}^{2}-2y\left(y_{m-1}-y_{m}\right)=r_{m-1}^{2}-r_{m}^{2} \end{array}\right.. \end{array}$$

We describe the system of equations in the above formula with *Ax* = *d* linear equations, which are

(6) $$\begin{array}{c} A=2\times \left[\begin{array}{cc} x_{1}-x_{m} & y_{1}-y_{m}\\ \vdots & \vdots \\ x_{m-1}-x_{m} & y_{m-1}-y_{m} \end{array}\right],d=\left[\begin{array}{c} r_{m}^{2}-r_{1}^{2}+x_{1}^{2}-x_{m}^{2}+y_{1}^{2}-y_{m}^{2}\\ \vdots \\ r_{m}^{2}-r_{m-1}^{2}+x_{m-1}^{2}-x_{m}^{2}+y_{m-1}^{2}-y_{m}^{2} \end{array}\right],x=\left[\begin{array}{c} x\\ y \end{array}\right]. \end{array}$$

Centroid algorithm:

(7) $$\begin{array}{c} \left(x,y\right)=\left[\frac{\left(x_{1}+x_{2}+x_{3}+x_{4}+x_{5}\right)}{5},\frac{\left(y_{1}+y_{2}+y_{3}+y_{4}+y_{5}\right)}{5}\right]. \end{array}$$

At present, with the mining and application of related network description signals, the theoretical transmission signal model construction is analyzed for different types of use. For the described signal transmission theory, it should mainly include the corresponding spatial propagation system and content, the reflection structure, and the model structure of the two-line ground. At the same time, for the path loss model, a distribution model should be established, and based on the existing log-constant distribution model, combined with the assumed signal propagation range in the signal model, highlight the concept of the circle in the ideal design as an analysis of the relevant node factors in the environmental impact factor. This analysis has certain volatility. However, compared with the actual transmission situation, it is carried out in the distribution model of logarithmic constant, which has better significance, can be widely used and in line with the actual situation.

The logarithmic-constant distribution model is shown in the following formula:

(8) $$\begin{array}{c} PL\left(d\right)\left[\text{d}B\right]=\bar{PL}\left(d_{0}\right)+X_{\sigma }=\bar{PL}\left(d_{0}\right)+10n\left(\frac{d}{d_{0}}\right)+X_{\sigma }. \end{array}$$

According to formula (8), the received power at each point can be obtained:

(9) $$\begin{array}{c} \text{RSSI}=P+G-PL\left(d\right)\left[\text{d}B\right]. \end{array}$$

After analysis, combined with the influence of factors such as temperature and humidity in the actual application environment, barometric obstacles, etc., during the launch process of the node, the refraction system used in the relevant transmission process will change, which will lead to environmental factors affecting the RSSI. The influence of the measured value gradually becomes larger and affects the signal reception.

(10) $$\begin{array}{c} \left\{\begin{array}{c} \sqrt{{\left(x_{0}-x_{a}\right)}^{2}+{\left(y_{0}-y_{a}\right)}^{2}}=r_{a}\\ \sqrt{{\left(x_{0}-x_{b}\right)}^{2}+{\left(y_{0}-y_{b}\right)}^{2}}=r_{b}\\ \sqrt{{\left(x_{0}-x_{c}\right)}^{2}+{\left(y_{0}-y_{c}\right)}^{2}}=r_{c} \end{array}\right.. \end{array}$$

The three-point centroid point is *D* which can be obtained by the following formula.

(11) $$\begin{array}{c} \left\{\begin{array}{c} \sqrt{{\left(x_{1}-x_{a}\right)}^{2}+{\left(y_{1}-y_{a}\right)}^{2}}=r_{a1}\\ \sqrt{{\left(x_{1}-x_{b}\right)}^{2}+{\left(y_{1}-y_{b}\right)}^{2}}=r_{b1}\\ \sqrt{{\left(x_{1}-x_{c}\right)}^{2}+{\left(y_{1}-y_{c}\right)}^{2}}=r_{c1} \end{array}\right.. \end{array}$$

For this quadratic equation, we will find that the amount of calculation will be very large when solving, as shown in the following formula.

(12) $$\begin{array}{c} \left\{\begin{array}{c} 2\left(x_{b}-x_{a}\right)x+2\left(y_{b}-y_{a}\right)y=d_{a1}^{2}-d_{b1}^{2}-x_{a}^{2}+x_{b}^{2}-y_{a}^{2}+y_{b}^{2}\\ 2\left(x_{b}-x_{c}\right)x+2\left(y_{b}-y_{c}\right)y=d_{c1}^{2}-d_{b1}^{2}-x_{c}^{2}+x_{b}^{2}-y_{c}^{2}+y_{b}^{2}\\ 2\left(x_{a}-x_{c}\right)x+2\left(y_{a}-y_{c}\right)y=d_{c1}^{2}-d_{a1}^{2}-x_{c}^{2}+x_{a}^{2}-y_{c}^{2}+y_{a}^{2} \end{array}\right.. \end{array}$$

The correction coefficient *θ* is obtained by the following formula.

(13) $$\begin{array}{c} \theta_{1}=1-\sqrt[{n}]{\frac{l_{a1}^{n}+l_{b1}^{n}+l_{c1}^{n}}{d_{a1}^{n}+d_{b1}^{n}+d_{c1}^{n}}}, \end{array}$$

(14) $$\begin{array}{c} \theta_{a1}=\theta_{1}\times \frac{3\times d_{a1}}{d_{a1}+d_{b1}+d_{c1}}. \end{array}$$

The corrected distance *d*_*a*2_ is obtained by formula (14), and *d*_*b*2_ and *d*_*c*2_ are calculated in the same way.

(15) $$\begin{array}{c} d_{a2}=d_{a1}\times \left(1-\theta_{a1}\right). \end{array}$$

The trilateral positioning method is the most basic source node positioning algorithm. The basic idea is that when the distance from an unknown node to at least 3 anchor nodes is known, the geometric characteristics of the intersection of three circles at one point can be used to calculate itself.

(16) $$\begin{array}{c} \left\{\begin{array}{c} \sqrt{{\left(x-x_{a}\right)}^{2}+{\left(x-x_{a}\right)}^{2}}=l_{a}\\ \sqrt{{\left(x-x_{b}\right)}^{2}+{\left(x-x_{b}\right)}^{2}}=l_{b}\\ \sqrt{{\left(x-x_{c}\right)}^{2}+{\left(x-x_{c}\right)}^{2}}=l_{c} \end{array}\right.. \end{array}$$

Calculating formula (17) shows that the coordinates of the unknown node *O* are

(17) $$\begin{array}{c} \left[\begin{array}{c} x\\ y \end{array}\right]={\left[\begin{array}{cc} 2\left(x_{a}-x_{c}\right) & 2\left(y_{a}-y_{c}\right)\\ 2\left(x_{b}-x_{c}\right) & 2\left(y_{b}-y_{c}\right) \end{array}\right]}^{-1}\left[\begin{array}{c} x_{a}^{2}-x_{c}^{2}+y_{a}^{2}-y_{c}^{2}+d_{c}^{2}-d_{a}^{2}\\ x_{b}^{2}-x_{c}^{2}+y_{b}^{2}-y_{c}^{2}+d_{c}^{2}-d_{b}^{2} \end{array}\right]. \end{array}$$

The trilateral positioning method requires the measured distance to be accurate, that is, the three circles must intersect at one point for the above equation to be established; otherwise, the three circles intersect in an area.

The core of the maximum likelihood estimation method is the idea of minimum mean square error, which uses mathematical principles to estimate the coordinates of unknown nodes.

(18) $$\begin{array}{c} \left\{\begin{array}{c} {\left(x-x_{1}\right)}^{2}+{\left(y-y_{1}\right)}^{2}=l_{1}^{2}\\ \vdots \\ {\left(x-x_{n}\right)}^{2}+{\left(y-y_{n}\right)}^{2}=l_{n}^{2} \end{array}\right.. \end{array}$$

Starting from the second line, each line is subtracted from the previous line to obtain

(19) $$\begin{array}{c} \left\{\begin{array}{c} x_{1}^{2}-x_{n}^{2}+2\left(x_{1}-x_{n}\right)x+y_{1}^{2}-y_{n}^{2}-2\left(y_{1}-y_{n}\right)y=l_{1}^{2}-l_{n}^{2}\\ x_{n-1}^{2}-x_{n}^{2}+2\left(x_{n-1}-x_{n}\right)x+y_{n-1}^{2}-y_{1}^{2}-2\left(y_{n-1}-y_{n}\right)y=l_{n-1}^{2}-l_{n}^{2} \end{array}\right.. \end{array}$$

Remember:

(20) $$\begin{array}{c} X=\left[\begin{array}{c} x\\ y \end{array}\right],\\ A=\left[\begin{array}{cc} 2\left(x_{1}-x_{n}\right) & 2\left(y_{1}-y_{n}\right)\\ \vdots & \vdots \\ 2\left(x_{n-1}-x_{n}\right) & 2\left(y_{n-1}-y_{n}\right) \end{array}\right],\\ B=\left[\begin{array}{c} x_{1}^{2}-x_{n}^{2}+y_{1}^{2}-y_{n}^{2}+l_{n}^{2}-l_{1}^{2}\\ \vdots \\ x_{n-1}^{2}-x_{n}^{2}+y_{n-1}^{2}-y_{n}^{2}+l_{n}^{2}-l_{n-1}^{2} \end{array}\right]. \end{array}$$

Then, the equation *AX*=*B* can be obtained, so

(21) $$\begin{array}{c} X={\left(A^{T}A\right)}^{-1}A^{T}B. \end{array}$$

These data packets contain key information such as the anchor node's node number and real geographic location.

(22) $$\begin{array}{c} \left(x_{m},y_{m}\right)=\left(\frac{\sum_{m=1}^{M}x_{m}}{M},\frac{\sum_{m=1}^{M}x_{y}}{M}\right). \end{array}$$

The original LRMD model can be expressed as

(23) $$\begin{array}{c} \underset{U,V}{\text{min}}{\left\|M-UV^{T}\right\|}_{\ell_{p}}. \end{array}$$

The original LRMD can be transformed into a matrix reconstruction model by adding an orthogonal projection operator, which can be expressed as

(24) $$\begin{array}{c} \underset{U,V}{\min}{\left\|P_{\Omega }\left(M-UV^{T}\right)\right\|}_{\ell_{p}}. \end{array}$$

It can be defined as:

(25) $$\begin{array}{c} {\left[P_{\Omega }\left(M\right)\right]}_{ij}=\left\{\begin{array}{cc} M_{ij}, & \left(i,j\right)\in \Omega \\ 0, & \text{otherwise } \end{array}\right.. \end{array}$$

### 3.4. Simulation Analysis

Based on the influence of relevant key factors in the wireless sensor, combined with the requirements, the rational construction of the relevant wireless sensor network technology based on node positioning is carried out.

1. Focus on reflecting the two-dimensional plane, conduct feasibility research on the problems related to self-localization under three-dimensional space conditions, and apply it to the current anchor node position and distance information.
2. For the node analysis of the sensor, it should be combined with the space electric wave propagation model in free space to realize a reasonable analysis of the communication system within the node range.
3. In the node structure of the corresponding sensor, using neighbor nodes can realize a reasonable evaluation of free communication.
4. For the sensor nodes, a reasonable construction of the communication radius should be realized through symmetrical communication capabilities, and all messages should be the focus to realize the analysis of related phenomena that can be correctly received in the end.

#### 3.4.1. Network Structure

*Communication Model*. Take the corresponding node as the basic information constructed by the communication model, combine it with its own center circle to analyze the communication radius *R* to form a communication capability based on anchor nodes, and then make the feasibility of sending and receiving related system content analysis.

**Figure 2:**
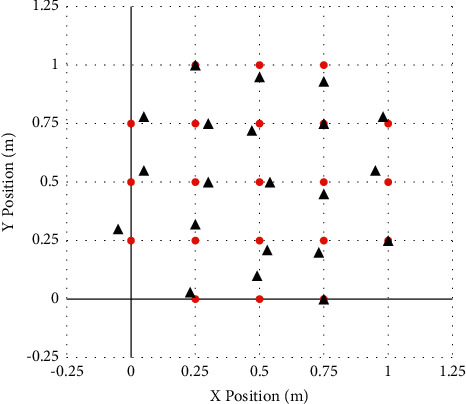
The first simulation of the RSSI algorithm.

**Figure 3:**
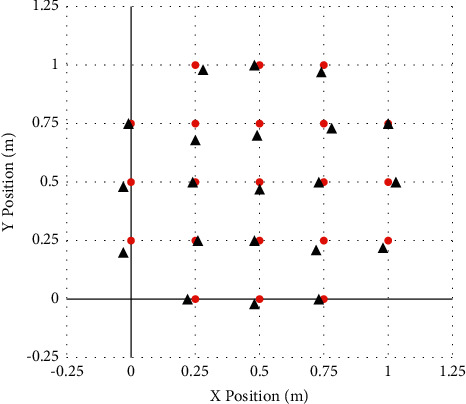
The second simulation of the RSSI algorithm.

**Figure 4:**
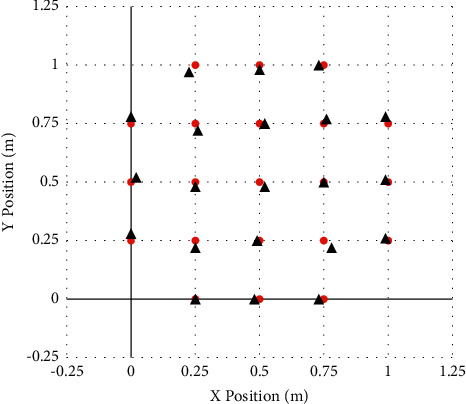
The third simulation of RSSI algorithm.

1. Use MATLAB to carry out calculation simulation, and choose 1 m × 1 m experiment field as the positioning area for experiment. By running the program, Figure 2 is obtained.
2. As shown in the figure above, we assume that 21 coordinate points are the original positions, and the red position in the figure is the original coordinate position. Then, we use the RSSI algorithm through simulation experiments to get the position measured by the distance. The position of the black circle in Figure 2 is the position measured by the algorithm.
3. The RSSI algorithm calculates and corrects twice continuously, and the following 2 simulation results can be obtained in succession, as shown in Figures 3 and 4.
4. The third simulation of the RSSI algorithm is shown in Figure 4.
5. After three simulations with different parameters (change of initial ranging position), the results are obtained.
6. It can be seen that every time the RSSI algorithm is corrected, the accuracy will increase once, that is, the position of the black circle and the position of the red circle are getting closer and closer.
7. Use MATLAB for algorithm simulation, choose a 100 m × 100 m experimental field as the positioning area, and establish a coordinate system and position definition (*x*, *y*), as shown in Table 1.

Unknown nodes are randomly distributed in the area, and the path loss coefficient is set to 2.4. Each simulation experiment is carried out 100 times, and the simulation results are the average of 100 times. The results of each simulation experiment are shown in Table 2.

Randomly arrange 200 sensor nodes in a 500*∗*500 WSN as a simulation site. During the experiment, the free space model is used. It is worth noting that the algorithm in this paper is not limited to the electromagnetic wave propagation model. As for the movement trajectory of the mobile anchor node, in order to reflect the generality, this article uses the random waypoint mode. Specifically, first, the mobile anchor node randomly selects a point outside the sensor monitoring area as the destination of this movement; second, the anchor node arbitrarily selects a rate that obeys a uniform distribution to reach the destination according to a straight line trajectory. When reaching the destination, the mobile anchor node arrives at the next destination at a different rate.

*Simulation Parameters*. This article selects experimental data based on the RFM TR3000 transmitter and stipulates that each packet has a length of 304 bits and a bandwidth of 64 kHz. Other parameters are shown in Table 3.

**Figure 5:**
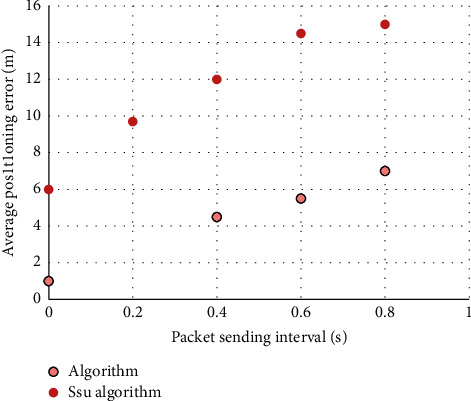
Comparison of average positioning errors under different packet sending intervals.

*Average Positioning Error and Packet Sending Interval*. The packet sending interval refers to the time between the mobile anchor node sending two adjacent packets. Under different packet sending intervals, compare the average positioning error in the non-ranging 3D positioning algorithm proposed in this paper and the SSU algorithm, as shown in Figure 5.

From the analysis of the above figure, it can be seen that as the packet sending interval decreases, the average positioning error of the two algorithms becomes correspondingly smaller. When the packet sending interval is 0.1 s, the average positioning error obtained by the non-ranging 3D positioning algorithm is less than 1 m, while the positioning error of the algorithm proposed by SSU is still greater than 5 m. Obviously, in the wireless channel environment, the algorithm proposed in this paper can work well in any wireless noise environment, and the performance of the SSU algorithm will be greatly reduced.

*Average Positioning Error and Anchor Node Moving Speed*. In the positioning system proposed by SSU, it believes that the product of moving speed and packet transmission interval should be constant. For comparison, in the simulation process, let the mobile anchor node send a packet at the same distance interval, such as (1 beacon/m). Therefore, while increasing the moving rate of the anchor node, the packet sending interval of the anchor node must be reduced, so as to maintain the original positioning error under different moving rates, as shown in Table 4.

It can be seen from Table 4 that as long as the product of the mobile anchor node's rate and the packet transmission interval remains unchanged, the positioning errors of the two algorithms will also remain at 0.8 m and 5.34 m, respectively. It can be seen that as the moving rate of the anchor node increases, the average execution time of the two algorithms will drop from 128 s to 51 s. This is because as the rate of the anchor node increases, more sensor nodes will receive the information sent by the mobile anchor node within a fixed period of time, which will inevitably reduce the average execution time of the entire network node. However, it is worth noting that the two algorithms only perform the beacon point selection algorithm when the single survival time of the mobile anchor node is exhausted, so there is no difference in the average execution time of the two algorithms.

**Figure 6:**
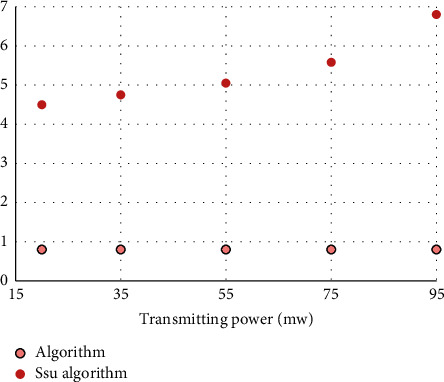
Comparison of average positioning errors under different transmission powers.

It can be seen that under the same average execution time, the performance of the positioning algorithm in this paper will be better than the positioning system proposed by SSU in terms of average positioning error and transmit power. This article uses five different mobile anchor node transmit powers, as shown in Figure 6.

It can be seen that as the transmit power increases, the performance of the SSU algorithm will be greatly reduced, and the average positioning error provided in this article will always remain at about 0.81. This is because in a wireless channel environment, the two types of beacon points obtained by the SSU algorithm will not be on the same circle, and at the same time, as the transmit power increases, the radius difference between the two circles will become larger. Compared with the algorithm proposed by SSU, both can obtain the beacon point correctly. Therefore, the positioning algorithm in this paper keeps the average positioning error at a constant under different transmission powers, while the average positioning error in the SSU algorithm will increase with the increase of the transmission power.

It can also be concluded from Table 5 that as the transmit power of the mobile anchor node increases, the average execution time of the two positioning algorithms will decrease. This is because if the transmit power of the mobile anchor node becomes larger, more sensor nodes will get the packets sent by the mobile anchor node in the same time. Therefore, sensor nodes will spend less time collecting beacon information.

## 4. Ink and Wash Animation VR Design and Its Application Based on Related Technology

### 4.1. Analysis of the Significance of Ink Animation VR Design

The rich Chinese culture and characteristic ink and wash elements are precious Chinese cultural heritage, condensing the essence of Chinese culture for thousands of years, and ink and wash animation is a dazzling pearl in the history of Chinese animation. At present, the emergence and rapid development of virtual reality provide new opportunities and possibilities for the formal expression of ink animation. It is a challenge to use 3D software technology and virtual reality platform to further develop traditional ink and wash animation and create a better and long-term animation work. We must think and explore in the wave of world cultural diversity. In this wave, we can make youth shine and maintain the vitality of culture and art ink.

### 4.2. Application Analysis of Ink Animation VR Design

The Chinese 3D computer ink and wash animation “Summer” was launched in 2003 by the original animation director Yingyi who studied Chinese painting. This work was selected as “SIGGRAPH 2003.” At that time, the conference video used computer technology and animation works, and its subtitle pictures were quoted, depicting the quiet pond in summer and the encounter of dragonflies, lotus, pond, and goldfish, which was full of poetry and painted with quiet ink.

White space is a unique composition and aesthetic feature of Chinese painting. In traditional two-dimensional animation, leaving white space repeatedly used to express meaning is a means of bleaching black. The summer pond is a unique method of white space. Like a lotus leaf floating on the water, the location of the water is depicted as invisible. The fish that compete and chase underwater not only represent the purity of the water but also enhance the dynamics of the water floating in the sky. In summer, only lotus flowers, rocks, old trees, and poets remain on the entire screen. By omitting redundant distant species, the composition is closer to the meaning of painting, giving people unlimited space for thought.

In addition, since the characters and scenes of the 3D animation are constructed in a three-dimensional shape, which is different from the plane motion effect of ordinary two-dimensional animations, all objects in summer have the perspective sense of three-dimensional space, and can swing to different angles in the script to shoot the camera. The sense of space and level are also very good.

### 4.3. Status Quo of Ink Animation VR Design

Animation is a special form of artistic expression, and its theme and content reflect the national culture. Today, with the increasingly obvious trend of globalization, culture is also going global in the wave of the times. In the process of importing Chinese animation to the world, ink and wash animation is the first choice to use traditional Chinese cultural elements to sublimate the theme animation and colors and to better understand and appreciate Chinese culture through animation. Chinese ink and wash animation is the expression that best reflects the characteristics of Chinese culture, and it is also the essence of Chinese manufacturing. The reason for the success of these classic ink and wash animation works is that it not only interprets the complex production methods but also interprets the unique visual language and culture of China.

With the rapid development of computer technology, digital animation was born. At the end of the 20th century, digital animation production technology became more and more complete, and it also provided a new development opportunity for ink and wash animation. With the support of the computer platform, the popularity of personal computer (PC) has led many people to the idea of making animations. Mobile movies that used to consume time and energy have disappeared from the stage of history. The emergence of digital animation solves the problem that the production time of traditional ink animation is much longer than that of other unit animations. Because the methods and processes of making animated digital ink are almost the same as those of other major digital animations, the software, usage, and technical means are also similar. This method of converting computer technology into animation production creates a condition for ink animation to surpass other animation creation in terms of efficiency. The redevelopment of ink and wash animation in the digital age provides strong security for survival and development in the fierce market competition in the future.

Digital animation production technology has not only become a catalyst for the development of ink animation but also adds a new language and visual expression method to ink animation. In the 3D ink animation, the stretch camera method and the translation of the traditional 2D ink animation have attracted people's attention. The ability of expressing space in three-dimensional manufacturing technology makes the flat ink have a sense of volume and perspective, and different angles can be exposed together with the walking lens. This is impossible to express in traditional ink and wash animations. These technological breakthroughs have expanded the expressive capabilities of 3D video inks and injected more energy into video inks.

Although ink animation in the digital age has many advantages, many internal and external factors still restrict the development of ink animation. These elements come from two directions. One of them is external factor, such as immature ink and wash animation technology, lack of professional production personnel, small production team, and the influence of other types of animation at home and abroad. On the other hand, there are internal factors such as subject restrictions, language browsing restrictions, and lack of entertainment.

### 4.4. Development Strategy of Ink Animation VR Design

There are many examples of foreign countries accepting Chinese traditional cultural elements. For example, “Kung Fu Panda” has become popular in the past few years. In this animated film, traditional Chinese cultural elements such as Kung Fu and Tai Chi are enriched and innovated in the animation and appear in new ways. Who said that only people can do kung fu? The Kung Fu World of martial arts panda is composed of various small animals with anthropomorphic characteristics; pandas can also become kung fu masters and save martial arts.

At present, in the process of animated films, in order to attract audiences and achieve higher box office, it is very important to produce high-precision details and animation effects of large scenes because they require better creative presentation, more wavy stories, and more powerful movie viewing effects. Compared with successful foreign animated films, the content is warm, and the black animation of poetry art is significantly inferior in dynamics. On the other hand, the soft and fuzzy visual experience and the spacious and profound narrative expression of ink and wash animation are the unique aesthetic characteristics of ink and wash animation, and this content creation method is the main factor restricting the development of ink and wash animation.

Traditional Chinese ink and wash animation stands out in the animation world with its fresh, patternless, empty, and quiet artistic expression. However, there have been many successful precedents, but later ink and wash animation has added artistic expression to weaken the expression of animation. Because ink animation is also a kind of animation, if we only pursue painting techniques, we ignore the fact that animation itself is subject-oriented and a visual expression. From the perspective of animation browsing language, Chinese animation ink painting still needs to learn from excellent foreign animation.

## 5. Conclusion

Ink and wash elements support China's 1,000-year cultural atmosphere. As a visual symbol, it has a strong traditional Chinese culture. The classic ink and wash animation created with ink and wash elements as visual symbols is a shining pearl in the history of animation in the world. The maturity and development of three-dimensional computer technology and virtual reality technology have brought new opportunities and challenges to ink and wash animation. Using computer three-dimensional technology to produce ink and wash animation is a collision between modern technology and traditional art. Another catalyst for the inheritance and development of Chinese ink painting and other traditional arts has been found. With the support of digital technology, ink and wash animated has made great progress in many aspects, such as the expressiveness of the picture, the expression of the characters' movements, and the browsing of language structures. It gives ink animation a new art form and is used in many fields such as TV packages, games, and new media. They have greatly improved the competitiveness of many types of ink and wash animations and are conducive to the long-term development and promotion of ink and wash animations. In the research on the creative methods of VR ink figure painting, the author applies various creative methods showing the changes of ink, fake fruit, and dryness to the three-dimensional ink production of image painting, and makes many attempts on the basis of the composition method of Chinese ink painting. People have revised the realization degree of ink effect in VR environment, and are committed to the production of VR virtual animation, that is, a world full of ink.

## Figures and Tables

**Table 1:** Position coordinates of 16 anchor nodes.

Serial number	Coordinate
1	(0, 0)
2	(33, 0)
3	(67, 0)
4	(100, 0)
5	(0, 33)
6	(33, 33)
7	(67, 33)
8	(100, 33)
9	(0, 67)
10	(33, 67)
11	(67, 67)
12	(100, 67)
13	(0, 100)
14	(33, 100)
15	(67, 100)
16	(100, 100)

**Table 2:** Simulation results.

Number of anchor nodes	Loss factor	Distance correction times	Positioning error (ill)
4	2.4	1	13.31
9	2.4	1	4.11
16	2.4	1	1.05
25	2.4	1	0.46
16	3.5	1	0.65
16	5	1	0.59
4	2.4	2	13.28
16	5	2	0.63
16	5	0	3.02
16	2.4	0	5.04
9	2.4	0	6.78

**Table 3:** Simulation parameters.

Parameter	Size
Packet sending interval (s)	0.1, 0.3, 0.5, 0.7, 0.9
Anchor knot survival time (s)	100
Maximum moving speed (m/s)	10, 20, 30, 40, 50
Threshold (in)	110, 130, 150, 170, 190
Transmitting power (mw)	15, 25, 45, 65, 85

**Table 4:** Comparison of positioning performance under different speeds.

Maximum moving speed (m/s)		10	20	30	40	50
Packet sending interval (s)		0.1	0.05	0.033	0.025	0.02
Average positioning error (in/)	Algorithm	0.82	0.81	0.81	0.80	0.80
	SSU algorithm	5.34	5.35	5.33	5.34	5.33

Average execution time (s)	Algorithm	128.16	75.63	65.35	57.26	50.65
	SSU algorithm	127.89	74.88	66.11	57.13	50.70

**Table 5:** Comparison of average execution time under different transmit power.

Transmit power (mw)	Average execution time (s)	
	Algorithm	SSU algorithm
15	172.34	171.98
25	150.36	150.23
45	128.16	127.89
65	100.79	101.74
85	95.26	95.65

## Data Availability

The data used to support the findings of this study are available from the corresponding author upon request.
